# Ferroptosis and Lipid Metabolism in Acute Myocardial Infarction

**DOI:** 10.31083/j.rcm2505149

**Published:** 2024-04-30

**Authors:** Xinyu Wu, Jingru Li, Huan Cheng, Luqiao Wang

**Affiliations:** ^1^Department of Cardiology, The First Affiliated Hospital of Kunming Medical University, 650032 Kunming, Yunnan, China

**Keywords:** ferroptosis, acute myocardial infarction, lipid peroxidation, lipid metabolism

## Abstract

Acute myocardial infarction (AMI) is triggered by the blockage of coronary arteries, leading to restricted blood flow to the myocardium, which results in damage and cell death. While the traditional understanding of cell death primarily revolves around apoptosis, a new player in the game has emerged: ferroptosis. This novel form of cell death relies on iron and is propelled by reactive oxygen species (ROS). Lipid metabolism, an indispensable physiological process, plays a vital role in preserving cellular homeostasis. However, when this metabolic pathway is disrupted, the accumulation of excess waste increases, specifically lipid peroxides, which are strongly linked to the occurrence and progression of AMI. As a result, comprehending this complex interaction between ferroptosis and lipid metabolism could pave the way for new therapeutic approaches in tackling AMI.

## 1. Introduction

Acute myocardial infarction (AMI), a subset of acute coronary syndrome, is a prevalent and serious disease affecting countless individuals across the globe. It is characterized by alarmingly high rates of morbidity, mortality, and recurrence, making it a major health concern worldwide [1, 2]. The primary culprit behind AMI is the formation of acute thrombus, AMI occurs when atherosclerotic plaques rupture, causing a sudden blockage of blood vessels. One of the well-established factors contributing to the occurrence and progression of AMI is abnormal blood lipid metabolism. For example, elevated plasma triglyceride levels also play a pivotal role in promoting microthrombus formation [3]. When the cholesterol efflux is impaired, plaques in the arteries can become unstable and prone to rupture, leading to the formation of acute thrombus and subsequent AMI [4]. In premenopausal women, high breast fat accumulation can produce and release inflammatory molecules implicit in vascular damage, atherosclerosis, leading to an increased risk of subsequent cardiovascular adverse events [5]. Lipid metabolism is of utmost importance in preserving a well-functioning cardiovascular system [6]. A study showed that metformin may reduce perimeter coronary fat levels by targeting the mediated-glucose cotransporter 2 (SGLT2)/leptin axis, improving adverse cardiovascular outcomes in diabetic patients [7]. Additionally, follow-ups of patients undergoing minimally invasive cardiopulmonary bypass found that long-term administration of sodium-glucose transporter 2 inhibitors could improve the adverse cardiovascular clinical outcomes by reducing the inflammatory burden [8]. In addition, an observational study showed that proprotein convertase subtilis protease-kexin 9 (PCSK9) inhibitors reduced cholesterol and blood lipids more significantly than other lipid-lowering drugs, providing effective cardiovascular benefits [9]. Furthermore, another significant mechanism involved in the pathogenesis of AMI is the increased production of reactive oxygen species (ROS) [10]. Interestingly, one study has shed light on the role of long non-coding RNA (lncRNA) lung cancer associated transcript 1(LUCAT1) in modulating ROS levels during AMI and its potential cardioprotective effects, acting through a competing endogenous RNA (ceRNA) mechanism [11].

The susceptibility of polyunsaturated fatty acid phospholipids (PUFA-PLs), which are found in cellular membranes, to peroxidation is the fundamental mechanism underlying ferroptosis. This vulnerability occurs when cells are exposed to high levels of iron and ROS [12]. This process involves a chain reaction of chemical reactions that give rise to the toxic accumulation of lipid peroxides within the cell membrane, ultimately compromising its integrity and triggering ferroptosis. Arachidonic acid (AA) and adrenic acid are the primary substrates involved in lipid peroxidation during ferroptosis [13]. The regulation of ferroptosis is heavily influenced by biological lipid metabolism, the related lipid metabolic processes include phospholipid peroxidation and other cellular events linked to this form of cell death [14]. This intricate interaction between lipid metabolism and ferroptosis holds significant potential for developing novel treatment strategies for AMI. This review briefly characterizes the interplay between ferroptosis and lipid metabolism and highlights the importance of this interplay in AMI.

## 2. Features of Ferroptosis

The term “ferroptosis” was introduced in 2012 to describe a unique iron-dependent form of cell death. Normally, polyunsaturated fatty acids (PUFAs) can be oxidized by lipoxygenase, but they are promptly reduced by the enzyme glutathione peroxidase 4 (GPX4) and its co-factor glutathione (GSH). However, when GPX4 is inhibited or GSH levels are depleted, lipid peroxides accumulate within cells, leading to cell death through a process known as ferroptosis [15]. Morphologically, cells undergoing ferroptosis display distinct changes: mitochondria become smaller, the mitochondrial cristae are reduced or disappear, and the mitochondrial membrane density is condensed. Despite these alterations, the nucleus remains intact. Biochemically characterized by disturbances in iron metabolism and elevated levels of lipid peroxidation [16]. Moreover, ferroptosis is genetically accompanied by specific changes in gene and protein expression. Notably, GPX4 is down-regulated, it is an enzyme tasked with attenuating lipid peroxidation [17], while acyl-CoA synthetase long-chain family member 4 (ACSL4), an enzyme involved in the synthesis of certain types of PUFAs, is up-regulated [18]. Overall, the distinct morphological, biochemical, and genetic features of ferroptosis provide a comprehensive understanding of this unique cell death pathway and differentiate it from other types of cell death.

Iron is an essential micronutrient in the human body, serving crucial roles in various systems such as the blood, digestive, and immune systems. To maintain iron balance, circulating iron binds to the transferrin receptor 1 (TFR1) on the cell membrane and is internalized into the cell through endocytosis. Within the cell, six-transmembrane epithelial antigen of prostate 3 (STEAP3) is responsible for reducing iron to its ferrous form before transferring it to the labile iron pool located in the cytoplasm [19]. The function of ferroportin (FPN) is to help prevent excess ferrous iron accumulation by converting it to ferric iron, thereby preserving iron homeostasis in the body [20]. However, when iron overload occurs or cells are damaged, the delicate balance of iron homeostasis is disrupted. This excessive ferrous iron can induce ferroptosis in cells, leading to cell death. Recent studies have unveiled a substantial connection between autophagy and ferroptosis. Specifically, the degradation of ferritin, a protein that stores iron, is a process involving autophagy. During this autophagic reaction, ferrous ions are released, increasing the levels of ferrous iron within cells and triggering ferroptosis [21, 22]. As a result, autophagy may play a role in triggering ferroptosis by affecting iron homeostasis in cardiomyocytes, thereby exacerbating cardiomyocyte injury.

## 3. Ferroptosis and Lipid Peroxidation in AMI

Imbalances in redox homeostasis are a common characteristic of many diseases [23]. When the body loses control over lipid peroxidation, lipid peroxidation is activated and executes ferroptosis [24]. In ferroptosis, lipid peroxidation primarily depends on the presence of excess ferrous ions, metabolite ROS, and phospholipids containing polyunsaturated fatty acid chains. Excess ferrous ions play a pivotal role in promoting lipid peroxidation. They facilitate the generation of ROS through a process known as the Fenton reaction and also activate specific enzymes like lipoxygenase, which further contribute to lipid peroxidation [25]. ROS themselves are a product of normal physiological processes in the body and serve essential functions in cell homeostasis and signal transduction. Various types of ROS exist, including superoxide anion radicals ($O_{2}$^•–^), hydroxyl radicals (•OH), as well as non-radical oxidants like hydrogen peroxide ($H_{2}$$O_{2}$) and singlet oxygen (^1^$O_{2}$) [26]. Oxidative stress occurs when the production of ROS exceeds the body’s antioxidant defense mechanisms, leading to an imbalance that results in cellular damage. Interestingly, research has identified a fascinating phenomenon called ROS-induced ROS release. The accumulation of ROS can trigger the opening of the mitochondrial permeability transition pore, leading to a larger-scale release of ROS from the mitochondria [27]. This feedback loop can further exacerbate oxidative stress and cellular damage, creating a vicious cycle of ROS production and release. Two primary sources contribute to the generation of ROS in cells. The first source is the mitochondrial respiratory chain. Reduced flavin yellow nucleotides in complex 1 of the respiratory chain can leak high-energy electrons to oxygen ($O_{2}$), resulting in the formation of ROS. The second source is macrophages, where nicotinamide adenine dinucleotide phosphate (NADPH) oxidase is a major contributor to ROS production [28]. PUFAs are highly susceptible to ROS-induced peroxidation. PUFA-PLs are particularly vulnerable to lipid peroxidation, and they serve as the main substrates for this process. Within the mitochondria, $H_{2}$$O_{2}$ produced by the respiratory chain can enter the mitochondrial membrane and be converted into a potent oxidant known as the hydroxyl radical. This hydroxyl radical then oxidizes polyunsaturated fatty acids in the mitochondrial membranes, leading to the formation of lipid hydroperoxides (ROOH) [29].

GPX4, the cornerstone of the body’s antioxidant defense, uses glutathione as a cofactor to reduce cytotoxic lipid peroxides to corresponding alcohols to terminate lipid peroxidation [30]. Therefore, by promoting the process of transcription and translation of GPX4, ferroptosis in myocardial infarction can be alleviated and exert a strong cardioprotective effect. G-rich RNA sequence binding factor 1 (Grsf1) is a mitochondrial RNA-binding protein that regulates the translation process of GPX4. Geniposide (GEN), a substance extracted from the traditional Chinese medicine Gardenia, can reverse myocardial infarction-induced oxidative stress and ferroptosis by targeting the Grsf1/GPX4 axis, and play a cardioprotective role [31]. Resveratrol has shown promising effects in alleviating myocardial injury induced by myocardial infarction, and recent research has shed light on its mechanism of action by targeting the Lysine(K) acetyltransferases 5 (KAT5)/GPX4 axis [32]. Additionally, researchers confirmed the interaction between lncRNA AC005332.7, microRNA (miR)-331-3p, and cyclin D2 (CCND2) by a series of experiments. Over-expression of AC005332.7 led to decreased levels of ROS, malondialdehyde, iron ions, and ACSL4 expression. Conversely, the expressions of GSH and GPX4 increased. These findings suggest that lncRNA AC005332.7 inhibits ferroptosis and alleviates AMI by regulating the miR-331-3p/CCND2 axis [33].

The transcription factor nuclear factor erythroid 2-related factor 2 (NRF2) is a critical regulator of cellular antioxidant responses [34], and plays a pivotal role in the “NRF2-lipid peroxidation-ferroptosis” axis, which is involved in ferroptosis in various diseases. NRF2 has been found to inhibit ferroptosis by downregulating heme oxygenase 1 (HO-1) [35, 36], HO-1 is responsible for decomposing heme into ferrous ions and certain enzymes that can induce ferroptosis, promoting lipid peroxidation [37]. NRF2 activation has been shown to have cardioprotective effects. A study showed the NRF2/HO-1 pathway can be activated by Icariin, inhibiting ferroptosis by reducing reactive oxygen species and lipid peroxidation in hypoxic/reoxygenated cardiomyocytes [38]. NRF2 also regulates lipid peroxidation by affecting various intermediate metabolites and enzymes involved in glutathione synthesis and metabolism [39]. For instance, NRF2 can modulate the activity of GPX4 and iron homeostasis by targeting solute carrier family seven member 11 (SLC7A11), contributing to ferroptosis resistance by reducing the level of lipid peroxidation in myocardial infarction [40]. Additionally, lncRNA metastasis-associated lung adenocarcinoma transcript 1 (MALAT1) has been found to be up-regulated in hypoxia/reoxygenation-treated cardiomyocytes and rat heart tissue. Inhibition of MALAT1 can increase the protein expression level of NRF2/SLC7A11 in myocardial infarction models, both *in vitro* and *in vivo*, while reducing ferrous ion levels and lipid peroxidation in cells. This implies that MALAT1 represents a promising target for the prevention and treatment of AMI [41, 42].

SLC7A11, a component of System Xc-, plays a crucial role in the generation of the antioxidant GSH. It transports extracellular cystine into the cell, where it is reduced to cysteine, a key component of GSH. GSH, in turn, helps reduce ROS under the action of glutathione reductase, contributing to cellular antioxidant defense [43, 44]. Ubiquitin-specific peptidase 22 (USP22), a member of the deubiquitinase family, possesses deubiquitinase activity and stabilizes sirtuin-1 (SIRT1). This stabilization of SIRT1 has been found to influence the acetylation level of P53, which, in turn, regulates the key protein SLC7A11 in the ferroptosis signaling pathway, contributing to cardiomyocyte resistance against ferroptosis [45]. Furthermore, myocardial infarction plasma exosomes have been implicated in the regulation of various pathological mechanisms after myocardial infarction. Experiments conducted by Li *et al*. [46] demonstrated that exosomes from patients with MI contained low levels of miR-26b-5p, which directly targeted the SLC7A11/GSH/GPX4 axis. This interaction mediated the effect of exosomes on ferroptosis, suggesting that the exosomes from patients with MI (MI-Exo)/miR-26b-5p/SLC7A11/GSH/GPX4 axis may represent a promising target for future treatments of AMI.

## 4. Ferroptosis and Lipid Metabolism in AMI

The control of lipid metabolism is of significant importance in influencing susceptibility to ferroptosis. PUFA-PLs, such as AA and adrenaline acid (AdA), are extremely prone to free radical oxidation mediated by lipoxygenases (ALOXs), leading to the generation of peroxidation products. This process disrupts the lipid bilayer and promotes ferroptosis [47]. Two important enzymes, Extralong-chain fatty acid protein 5 (ELOVL5) and fatty acid desaturase 1 (FADS1), regulate the biosynthesis of AA and AdA. Depletion of ELOVL5 and FADS1 can lead to decreased lipid peroxidation and increased resistance to ferroptosis [48]. Arachidonic acid can be esterified to AA-CoA by acyl-CoA synthetase long-chain family member 4 (ACSL4) and further processed into lysophospholipids (LysoPLs), which are prone to peroxidation in the presence of iron and ROS [49]. However, Fas-associated factor 1 (FAF1) exerts a protective function in ferroptosis by directly engaging with unbound arachidonic acid via its upstream activating sequence (UAS) domain, sequestering arachidonic acid, and preventing ${\mathrm{Fe}}^{2+}$-mediated fatty acid peroxidation [50]. Linoleic acid (LA), containing only one diallyl group, is the primary polyunsaturated fatty acid in plant food sources. It can be converted into arachidonic acid by the fatty acid desaturase FADS2 [51]. Monounsaturated fatty acids (MUFAs) have been shown to effectively block ferroptosis by competing with PUFAs for phospholipid production [52]. Dietary levels of MUFAs and PUFAs significantly impact cellular susceptibility to ferroptosis [53].

Excess cholesterol has been shown to inhibit the ferroptosis signaling pathway, influencing cellular susceptibility to ferroptosis. For instance, the circulating cholesterol metabolite 27-hydroxycholesterol (27HC) confers ferroptosis resistance in cells, and activation of 7-dehydrocholesterol reductase in cholesterol biosynthesis can also increase ferroptosis resistance [54]. Under metabolic stress conditions characterized by adenosine triphosphate (ATP) depletion, the activity of the energy-sensing kinase adenosine monophosphate-activated protein kinase (AMPK) is activated. This, in turn, inhibits acetyl-CoA carboxylase (ACC), which is responsible for converting acetyl-CoA to malonyl-CoA and reduces the levels of PUFA-PLs, ultimately leading to the blocking of ferroptosis [55]. The enzyme ACSL4 significantly contributes to the conversion of arachidonic acid and adrenaline acid to phosphatidylethanolamine (PE), which is highly susceptible to peroxidation. In conditions rich in iron and ROS, this process induces ferroptosis [56]. In the hypoxia/reoxygenation myocardial injury model, lncAABR07025387.1 expression was increased, and it was found to up-regulate the expression of the ferroptosis-promoting factor ACSL4 by acting as a sponge for miR-205. This created a ceRNA (competing endogenous RNA) network related to ferroptosis in myocardial infarction, involving lncRNA AABR07025387.1, miR-205, and ACSL4. This network is enriched in mechanisms of non-coding RNAs regulating ferroptosis in the context of myocardial infarction [57]. In an AMI model, lncRNA Gm47283/miR-706/prostaglandin-endoperoxide synthase 2 (Ptgs2) regulatory axis promotes the form of the peroxidation of PUFAs through activating the downstream signal molecule lipoxygenase 15 (ALOX15) [58]. Understanding the intricate regulatory mechanisms of ferroptosis and the involvement of cholesterol, non-coding RNAs, and other factors provides valuable information for pinpointing potential therapeutic targets for modulating ferroptosis and safeguarding against conditions like AMI.

## 5. Conclusions and Perspectives

**Fig. 1.**
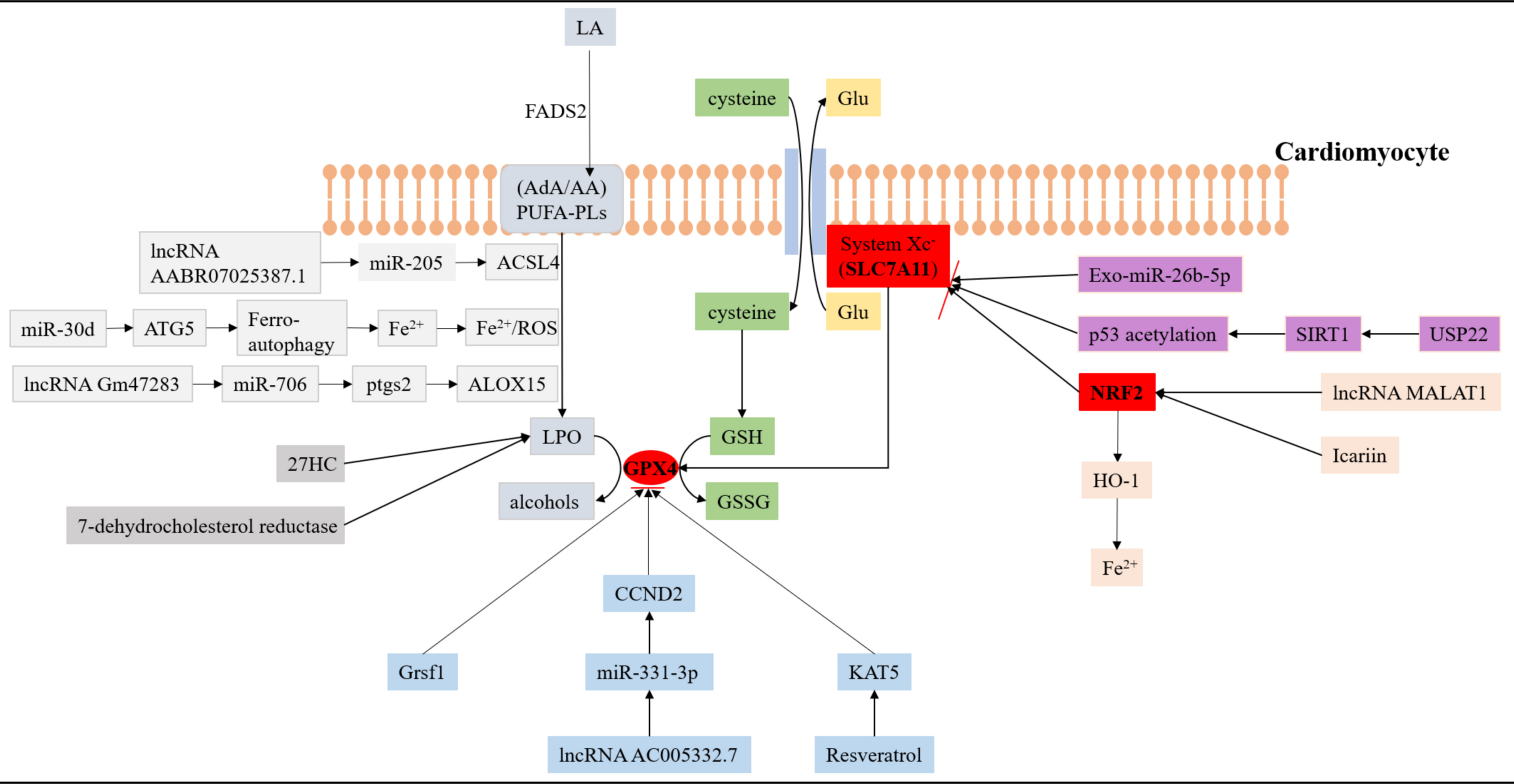
**The interaction of ferroptosis and lipid metabolism in acute myocardial infarction**. SLC7A11, GPX4 and NRF2 are three key antioxidant molecules that regulate the pathological process of ferroptosis in AMI. Many non-coding RNAs, biomolecules, and traditional Chinese medicines can affect the level of lipid peroxidation in cardiomyocytes by participating in the regulation of antioxidant molecules. (1) The pathways marked by the light grey background represent relevant processes regulating lipid peroxidation. (2) The pathway marked by the blue background represents the related process of regulating the antioxidant molecule GPX4. (3) The pathway marked by the pink background represents a related process of regulating the antioxidant molecule NRF2. (4) The pathway marked by the purple background represents a related process of regulating the antioxidant molecule SLC7A11. GPX4, glutathione peroxidase 4; NRF2, nuclear factor-erythroid factor 2-related factor 2; AMI, acute myocardial infarction; LA, linoleic acid; FADS2, a kind of fatty acid desat urase; AA, arachidonic acid; AdA, Adrenoic acid; PUFA-PLs, phospholipid-containing polyunsaturated fatty acids; LPO, lipid peroxidation; ACSL4, acyl-CoA synthetase long-chain family member 4; GSH, glutathione; ATG5, autophagy-related gene 5; Grsf1, a mitochondrial RNA-binding protein; CCND2, cyclin D2; 27HC, 27-hydroxycholesterol; USP22, ubiquitin specific peptidase 22; SIRT1, sirtuin-1; HO-1, heme oxy genase 1; ALOX15, lipoxygenase 15; Ptgs2, prostaglandin-endoperoxide synthase 2; ROS, reactive oxygen species; KAT5, K (lysine) acetyltransferase 5; GSSG, glutathione disulfide; Glu, glutamate; SLC7A11, solute carrier family 7a member 11; miR, microRNA; IncRNA, long non-coding RNA.

Our review has highlighted the intricate and inextricable link between lipid metabolism, ferroptosis, and AMI. Abnormal lipid metabolism and the accumulation of waste products play a significant role in the occurrence and progression of AMI. Ferroptosis, characterized by lipid peroxidation, is a pivotal player in the regulation of myocardial injury and maintains a close interaction with the body’s lipid metabolism (Fig. 1). In the context of ferroptosis, key anti-lipid peroxidation molecules such as GPX4, NRF2, and SLC7A11 play critical roles. Traditional Chinese medicines, active molecules, and non-coding RNAs have been identified as potential mediators of these anti-lipid peroxidation molecules, enriching the regulatory mechanism of ferroptosis in AMI. These active medicines and biomolecules against lipid peroxidation will be most possibly used to prevent and treat AMI in the near future. Notably, arachidonic acid and adrenaline acid are indispensable substrates in the lipid peroxidation pathway of ferroptosis, subject to regulation by a range of enzymes and signaling pathways. Cholesterol and its metabolites have also been shown to inhibit signaling pathways related to ferroptosis lipid metabolism and exert a cardioprotective role. So some lipid intake and their metabolism-related products can affect lipid peroxidation substrate/enzyme levels, and exert myocardial protective effects by regulating ferroptosis in AMI. Autophagic degradation of ferritin has been found to promote AMI by producing ferrous ions and mediating GPX4. The continuous study of medicines, active biomolecules, and the intake of some lipids will be very likely to develop into effective strategies for the prevention and treatment of AMI. In addition, necrosis is the predominant form of cardiomyocyte death after AMI. However, Tu *et al*. [59] showed that the combination of necrosis inhibitor (ponatinib) and ferroptosis inhibitor (deferoxamine) could better reduce ischemic heart injury *in vitro* and *in vivo* studies, this suggested a possible synergistic regulatory mechanism between ferroptosis and necrosis. Myocardial injury is a complex pathological process that needs to be considered in many ways, and drug development should be comprehensive to reflect this.

In our review, there are several studies that show ferroptosis in ischemia-reperfusion injury is closely related to lipid metabolism. In both *in vivo* and *in vitro* models, the activation of NRF2, lncRNAs (lncRNA MALAT1 and lncRNA AABR07025387.1) and USP22 can regulate ischemia-reperfusion injury by regulating lipid peroxidation of ferroptosis. It is highly likely that these targets will be used clinically to prevent and treat ischemia-reperfusion injury in the future. Therefore, as the most common and serious complication after restoring blood supply in AMI, future studies on ferroptosis and lipid metabolism in ischemia-reperfusion injury are warranted.

Moreover, changes in cardiac function and structure after myocardial infarction will lead to ventricular remodeling, which seriously affects the prognosis of patients with AMI. Previous studies found that the inhibition of ferroptosis could also alleviate ventricular remodeling after myocardial infarction. For example, Komai *et al*. [60] found that ferroptosis is associated with the inflammatory response during ventricular remodeling after AMI. Inhibition of inflammation and ferroptosis delays the extent of ventricular remodeling after myocardial infarction. Another study also showed that a marine-derived antioxidant reduces oxidative stress after AMI, thus exerting a protective role in ventricular remodeling and chronic heart failure after AMI. This natural antioxidant can play a protective role in ventricular remodeling after AMI by inhibiting ferroptosis [61]. The above studies suggest an inevitable link between ventricular remodeling after AMI and ferroptosis. Therefore, future continuous studies on ferroptosis and ventricular remodeling after AMI will be very likely to develop effective strategies for the prevention and treatment of ventricular remodeling after AMI.

In conclusion, the interaction between ferroptosis and lipid metabolism offers a potential therapeutic strategy for the treatment of AMI. However, many detailed mechanisms in this relationship require further study to fully comprehend the complexities of this association. Continued research in this area may lead to the development of novel and effective treatment approaches for AMI, ultimately improving patient outcomes and addressing the global burden of cardiovascular diseases.

## Abbreviations

LA, linoleic acid; FADS2, a kind of fatty acid desaturase; AA, arachidonic acid; AdA, Adrenoic acid; PUFA-PLs, phospholipid-containing polyunsaturated fatty acids; LPO, lipid peroxidation; GPX4, glutathione peroxidase 4; ACSL4, acyl-CoA synthetase long-chain family member 4; GSH, glutathione; ATG5, autophagy-related gene 5; Grsf1, a mitochondrial RNA-binding protein; CCND2, cyclin D2; 27HC, 27-hydroxycholesterol; USP22, ubiquitin-specific peptidase 22; SIRT1, sirtuin-1; NRF2, nuclear factor-erythroid factor 2-related factor 2; HO-1, heme oxygenase 1. ALOX15, lipoxygenase 15; Ptgs2, prostaglandin-endoperoxide synthase 2.

## References

[1] Li J, Wu X, Ma H, Sun G, Ding P, Lu S (2022). New developments in non-exosomal and exosomal ncRNAs in coronary artery disease. *Epigenomics*.

[2] Li J, Ma H, Wu X, Sun G, Yang P, Peng Y (2023). Promising roles of non-exosomal and exosomal non-coding RNAs in the regulatory mechanism and as diagnostic biomarkers in myocardial infarction. *Journal of Zhejiang University. Science. B*.

[3] Li T, Li X, Meng H, Chen L, Meng F (2020). ACSL1 affects Triglyceride Levels through the PPARγ Pathway. *International Journal of Medical Sciences*.

[4] Kingwell BA, Nicholls SJ, Velkoska E, Didichenko SA, Duffy D, Korjian S (2022). Antiatherosclerotic Effects of CSL112 Mediated by Enhanced Cholesterol Efflux Capacity. *Journal of the American Heart Association*.

[5] Sardu C, Gatta G, Pieretti G, Viola L, Sacra C, Di Grezia G (2021). Pre-Menopausal Breast Fat Density Might Predict MACE During 10 Years of Follow-Up: The BRECARD Study. *JACC. Cardiovascular Imaging*.

[6] Liu ZY, Liu F, Cao Y, Peng SL, Pan HW, Hong XQ (2023). ACSL1, CH25H, GPCPD1, and PLA2G12A as the potential lipid-related diagnostic biomarkers of acute myocardial infarction. *Aging*.

[7] Sardu C, D’Onofrio N, Torella M, Portoghese M, Mureddu S, Loreni F (2021). Metformin Therapy Effects on the Expression of Sodium-Glucose Cotransporter 2, Leptin, and SIRT6 Levels in Pericoronary Fat Excised from Pre-Diabetic Patients with Acute Myocardial Infarction. *Biomedicines*.

[8] Sardu C, Massetti M, Testa N, Martino LD, Castellano G, Turriziani F (2021). Effects of Sodium-Glucose Transporter 2 Inhibitors (SGLT2-I) in Patients With Ischemic Heart Disease (IHD) Treated by Coronary Artery Bypass Grafting *via* MiECC: Inflammatory Burden, and Clinical Outcomes at 5 Years of Follow-Up. *Frontiers in Pharmacology*.

[9] Marfella R, Prattichizzo F, Sardu C, Paolisso P, D’Onofrio N, Scisciola L (2023). Evidence of an anti-inflammatory effect of PCSK9 inhibitors within the human atherosclerotic plaque. *Atherosclerosis*.

[10] Su Q, Liu Y, Lv XW, Dai RX, Yang XH, Kong BH (2020). LncRNA TUG1 mediates ischemic myocardial injury by targeting miR-132-3p/HDAC3 axis. *American Journal of Physiology. Heart and Circulatory Physiology*.

[11] Xiao SH, Wang Y, Cao X, Su Z (2021). Long non-coding RNA LUCAT1 inhibits myocardial oxidative stress and apoptosis after myocardial infarction via targeting microRNA-181a-5p. *Bioengineered*.

[12] Sun Y, Chen P, Zhai B, Zhang M, Xiang Y, Fang J (2020). The emerging role of ferroptosis in inflammation. *Biomedicine & Pharmacotherapy*.

[13] Tang D, Chen X, Kang R, Kroemer G (2021). Ferroptosis: molecular mechanisms and health implications. *Cell Research*.

[14] Liang D, Minikes AM, Jiang X (2022). Ferroptosis at the intersection of lipid metabolism and cellular signaling. *Molecular Cell*.

[15] Lee JY, Nam M, Son HY, Hyun K, Jang SY, Kim JW (2020). Polyunsaturated fatty acid biosynthesis pathway determines ferroptosis sensitivity in gastric cancer. *Proceedings of the National Academy of Sciences of the United States of America*.

[16] Mou Y, Wang J, Wu J, He D, Zhang C, Duan C (2019). Ferroptosis, a new form of cell death: opportunities and challenges in cancer. *Journal of Hematology & Oncology*.

[17] Friedmann Angeli JP, Schneider M, Proneth B, Tyurina YY, Tyurin VA, Hammond VJ (2014). Inactivation of the ferroptosis regulator Gpx4 triggers acute renal failure in mice. *Nature Cell Biology*.

[18] Cui Y, Zhang Y, Zhao X, Shao L, Liu G, Sun C (2021). ACSL4 exacerbates ischemic stroke by promoting ferroptosis-induced brain injury and neuroinflammation. *Brain, Behavior, and Immunity*.

[19] Liu P, Wang W, Li Z, Li Y, Yu X, Tu J (2022). Ferroptosis: A New Regulatory Mechanism in Osteoporosis. *Oxidative Medicine and Cellular Longevity*.

[20] Li J, Cao F, Yin HL, Huang ZJ, Lin ZT, Mao N (2020). Ferroptosis: past, present and future. *Cell Death & Disease*.

[21] Liu J, Kuang F, Kroemer G, Klionsky DJ, Kang R, Tang D (2020). Autophagy-Dependent Ferroptosis: Machinery and Regulation. *Cell Chemical Biology*.

[22] Tang S, Wang Y, Ma T, Lu S, Huang K, Li Q (2020). MiR-30d inhibits cardiomyocytes autophagy promoting ferroptosis after myocardial infarction. *Panminerva Medica*.

[23] Gaschler MM, Stockwell BR (2017). Lipid peroxidation in cell death. *Biochemical and Biophysical Research Communications*.

[24] Ursini F, Maiorino M (2020). Lipid peroxidation and ferroptosis: The role of GSH and GPx4. *Free Radical Biology & Medicine*.

[25] Lei G, Zhuang L, Gan B (2022). Targeting ferroptosis as a vulnerability in cancer. *Nature Reviews. Cancer*.

[26] Su LJ, Zhang JH, Gomez H, Murugan R, Hong X, Xu D (2019). Reactive Oxygen Species-Induced Lipid Peroxidation in Apoptosis, Autophagy, and Ferroptosis. *Oxidative Medicine and Cellular Longevity*.

[27] Zorov DB, Juhaszova M, Sollott SJ (2014). Mitochondrial reactive oxygen species (ROS) and ROS-induced ROS release. *Physiological Reviews*.

[28] Herb M, Schramm M (2021). Functions of ROS in Macrophages and Antimicrobial Immunity. *Antioxidants (Basel, Switzerland)*.

[29] Zhou Q, Li T, Qin Q, Huang X, Wang Y (2022). Ferroptosis in lymphoma: Emerging mechanisms and a novel therapeutic approach. *Frontiers in Genetics*.

[30] Bai T, Li M, Liu Y, Qiao Z, Wang Z (2020). Inhibition of ferroptosis alleviates atherosclerosis through attenuating lipid peroxidation and endothelial dysfunction in mouse aortic endothelial cell. *Free Radical Biology & Medicine*.

[31] Shen Y, Wang X, Shen X, Wang Y, Wang S, Zhang Y (2022). Geniposide Possesses the Protective Effect on Myocardial Injury by Inhibiting Oxidative Stress and Ferroptosis *via* Activation of the Grsf1/GPx4 Axis. *Frontiers in Pharmacology*.

[32] Liu J, Zhang M, Qin C, Wang Z, Chen J, Wang R (2022). Resveratrol Attenuate Myocardial Injury by Inhibiting Ferroptosis *Via* Inducing KAT5/GPX4 in Myocardial Infarction. *Frontiers in Pharmacology*.

[33] Dai R, Yang X, He W, Su Q, Deng X, Li J (2023). LncRNA AC005332.7 Inhibited Ferroptosis to Alleviate Acute Myocardial Infarction Through Regulating miR-331-3p/CCND2 Axis. *Korean Circulation Journal*.

[34] Shen Y, Shen X, Wang S, Zhang Y, Wang Y, Ding Y (2022). Protective effects of Salvianolic acid B on rat ferroptosis in myocardial infarction through upregulating the Nrf2 signaling pathway. *International Immunopharmacology*.

[35] Li J, Lu K, Sun F, Tan S, Zhang X, Sheng W (2021). Panaxydol attenuates ferroptosis against LPS-induced acute lung injury in mice by Keap1-Nrf2/HO-1 pathway. *Journal of Translational Medicine*.

[36] Luo L, Huang F, Zhong S, Ding R, Su J, Li X (2022). Astaxanthin attenuates ferroptosis via Keap1-Nrf2/HO-1 signaling pathways in LPS-induced acute lung injury. *Life Sciences*.

[37] Liang J, Cao Y, He M, Li W, Huang G, Ma T (2021). AKR1C3 and Its Transcription Factor HOXB4 Are Promising Diagnostic Biomarkers for Acute Myocardial Infarction. *Frontiers in Cardiovascular Medicine*.

[38] Liu XJ, Lv YF, Cui WZ, Li Y, Liu Y, Xue YT (2021). Icariin inhibits hypoxia/reoxygenation-induced ferroptosis of cardiomyocytes via regulation of the Nrf2/HO-1 signaling pathway. *FEBS Open Bio*.

[39] Dodson M, Castro-Portuguez R, Zhang DD (2019). NRF2 plays a critical role in mitigating lipid peroxidation and ferroptosis. *Redox Biology*.

[40] Xu S, Wu B, Zhong B, Lin L, Ding Y, Jin X (2021). Naringenin alleviates myocardial ischemia/reperfusion injury by regulating the nuclear factor-erythroid factor 2-related factor 2 (Nrf2) /System xc-/ glutathione peroxidase 4 (GPX4) axis to inhibit ferroptosis. *Bioengineered*.

[41] Jiang YQ, Yang XY, Duan DQ, Zhang YY, Li NS, Tang LJ (2023). Inhibition of MALT1 reduces ferroptosis in rat hearts following ischemia/reperfusion via enhancing the Nrf2/SLC7A11 pathway. *European Journal of Pharmacology*.

[42] Wu X, Li J, Sun G, Yang J, Peng Y, Bai X (2023). Role of LncRNAs in the Pathogenesis of Coronary Artery Disease. *Reviews in Cardiovascular Medicine*.

[43] Wang L, Liu Y, Du T, Yang H, Lei L, Guo M (2020). ATF3 promotes erastin-induced ferroptosis by suppressing system Xc. *Cell Death and Differentiation*.

[44] Zhang W, Gong M, Zhang W, Mo J, Zhang S, Zhu Z (2022). Correction: Thiostrepton induces ferroptosis in pancreatic cancer cells through STAT3/GPX4 signalling. *Cell Death & Disease*.

[45] Ma S, Sun L, Wu W, Wu J, Sun Z, Ren J (2020). USP22 Protects Against Myocardial Ischemia-Reperfusion Injury via the SIRT1-p53/SLC7A11-Dependent Inhibition of Ferroptosis-Induced Cardiomyocyte Death. *Frontiers in Physiology*.

[46] Li H, Ding J, Liu W, Wang X, Feng Y, Guan H (2023). Plasma exosomes from patients with acute myocardial infarction alleviate myocardial injury by inhibiting ferroptosis through miR-26b-5p/SLC7A11 axis. *Life Sciences*.

[47] Tang D, Kroemer G (2020). Ferroptosis. *Current Biology: CB*.

[48] Jiang X, Stockwell BR, Conrad M (2021). Ferroptosis: mechanisms, biology and role in disease. *Nature Reviews. Molecular Cell Biology*.

[49] Chen X, Kang R, Kroemer G, Tang D (2021). Ferroptosis in infection, inflammation, and immunity. *The Journal of Experimental Medicine*.

[50] Cui S, Simmons G, Vale G, Deng Y, Kim J, Kim H (2022). FAF1 blocks ferroptosis by inhibiting peroxidation of polyunsaturated fatty acids. *Proceedings of the National Academy of Sciences of the United States of America*.

[51] Szczuko M, Kikut J, Komorniak N, Bilicki J, Celewicz Z, Ziętek M (2020). The Role of Arachidonic and Linoleic Acid Derivatives in Pathological Pregnancies and the Human Reproduction Process. *International Journal of Molecular Sciences*.

[52] Rodencal J, Dixon SJ (2023). A tale of two lipids: Lipid unsaturation commands ferroptosis sensitivity. *Proteomics*.

[53] Qi Y, Zhang X, Wu Z, Tian M, Chen F, Guan W (2022). Ferroptosis Regulation by Nutrient Signalling. *Nutrition Research Reviews*.

[54] Liu W, Chakraborty B, Safi R, Kazmin D, Chang CY, McDonnell DP (2021). Dysregulated cholesterol homeostasis results in resistance to ferroptosis increasing tumorigenicity and metastasis in cancer. *Nature Communications*.

[55] Lei G, Mao C, Yan Y, Zhuang L, Gan B (2021). Ferroptosis, radiotherapy, and combination therapeutic strategies. *Protein & Cell*.

[56] Li Y, Feng D, Wang Z, Zhao Y, Sun R, Tian D (2019). Ischemia-induced ACSL4 activation contributes to ferroptosis-mediated tissue injury in intestinal ischemia/reperfusion. *Cell Death and Differentiation*.

[57] Sun W, Wu X, Yu P, Zhang Q, Shen L, Chen J (2022). LncAABR07025387.1 Enhances Myocardial Ischemia/Reperfusion Injury *Via* miR-205/ACSL4-Mediated Ferroptosis. *Frontiers in Cell and Developmental Biology*.

[58] Gao F, Zhao Y, Zhang B, Xiao C, Sun Z, Gao Y (2022). Suppression of lncRNA *Gm47283* attenuates myocardial infarction via *miR-706*/ *Ptgs2*/ferroptosis axis. *Bioengineered*.

[59] Tu H, Zhou YJ, Tang LJ, Xiong XM, Zhang XJ, Ali Sheikh MS (2021). Combination of ponatinib with deferoxamine synergistically mitigates ischemic heart injury via simultaneous prevention of necroptosis and ferroptosis. *European Journal of Pharmacology*.

[60] Komai K, Kawasaki NK, Higa JK, Matsui T (2022). The Role of Ferroptosis in Adverse Left Ventricular Remodeling Following Acute Myocardial Infarction. *Cells*.

[61] Tang X, Nishimura A, Ariyoshi K, Nishiyama K, Kato Y, Vasileva EA (2023). Echinochrome Prevents Sulfide Catabolism-Associated Chronic Heart Failure after Myocardial Infarction in Mice. *Marine Drugs*.

